# Evaluation of Six eGFR Equations in Predicting Acute Kidney Injury in Patients after Off-Pump Coronary Artery Bypass Grafting: A Case Control Study

**DOI:** 10.31083/j.rcm2504120

**Published:** 2024-03-28

**Authors:** Jiwen Tang, Congcong Zhang, Weiwei Hu, Weili Qu

**Affiliations:** ^1^Department of Cardiac Surgical Care Unit, Affiliated Hospital of Qingdao University, 266003 Qingdao, Shandong, China; ^2^Department of Thoracic Surgery, Affiliated Hospital of Qingdao University, 266003 Qingdao, Shandong, China

**Keywords:** estimated glomerular filtration rate, chronic kidney disease-epidemiology equation, full age spectrum equation, acute kidney injury, coronary artery bypass grafting

## Abstract

**Background:**

There are six widely used equations to calculate the estimated glomerular filtration rate (eGFR) of patients. We aimed to assess the predictive power of preoperative eGFR calculated by these equations for the occurrence of postoperative acute kidney injury (AKI).

**Methods:**

Patients who underwent isolated coronary surgery from January 2016 to January 2021 were continuously enrolled. Serum creatinine and cystatin C used to calculate eGFR were both measured within 1 week before surgery. The eGFR was calculated using six equations: Cockcroft Gault (CG) equation, Chinese abbreviated modification of diet in renal disease (MDRD) equation, chronic kidney disease-epidemiology (CKD-EPI) equation, and full age spectrum (FAS) equation. Postoperative AKI was diagnosed by Kidney Disease Improving Global Outcomes criteria (KDIGO) (① urine volume <0.5 mL/kg/h for 6 h; ② an increase in serum creatinine by ≥26.5 µmol/L within 48 h; ③ an increase in serum creatinine to ≥1.5 times baseline levels, which is known or presumed to have occurred within the prior 7 days), and the occurrence of AKI within 7 days after surgery was followed.

**Results:**

A total of 1428 patients were included, of which 319 patients (25.5%) developed postoperative AKI. After adjustment, all eGFRs (CG OR = 0.983, MDRD OR = 0.983, ${\mathrm{CKD-EPI}}_{\text{crea}}$ OR = 0.97, ${\mathrm{CKD-EPI}}_{\text{cys}}$ OR = 0.955, ${\mathrm{FAS}}_{\text{crea}}$ OR = 0.978, ${\mathrm{FAS}}_{\text{cys}}$ OR = 0. 941, all *p* < 0.001) were significantly associated with AKI. The area under the receiver operating characteristic curve (AUC) was 0.621 for CG, 0.614 for MDRD, 0.643 for ${\mathrm{CKD-EPI}}_{\text{crea}}$, 0.739 for ${\mathrm{CKD-EPI}}_{\text{cys}}$, 0.643 for ${\mathrm{FAS}}_{\text{crea}}$, 0.744 for ${\mathrm{FAS}}_{\text{cys}}$, respectively. There was no difference in predictive power between ${\mathrm{FAS}}_{\text{cys}}$ and ${\mathrm{CKD-EPI}}_{\text{cys}}$ (*p* = 0.33, DeLong’s test).

**Conclusions:**

Preoperative eGFR calculated by ${\mathrm{FAS}}_{\text{cys}}$ and ${\mathrm{CKD-EPI}}_{\text{cys}}$ equations have better performance in predicting AKI after off-pump coronary artery bypass grafting than other equations.

## 1. Introduction

Postoperative acute kidney injury (AKI) is a common complication after heart surgery, with an incidence of between 5% and 42%. Postoperative AKI is associated with a variety of adverse events, such as prolonged intensive care unit (ICU) stay and increased mortality [1, 2]. The development of AKI is influenced by many clinical factors, such as advanced age, congestive heart failure, hyperglycemia, pre-existing kidney disease, and emergency surgery [1, 2]. Therefore, adequate assessment of preoperative renal function is particularly important for identifying high-risk patients and predicting postoperative AKI.

Estimated glomerular filtration rate (eGFR) is a convenient tool for assessing renal function in patients. In addition to the commonly used Cockcroft Gault (CG) equation [3], the modification of diet in renal disease (MDRD) equation [4], and chronic kidney disease-epidemiology (CKD-EPI) equation [5], there are also the Schwartz equation [6] for children and the Berlin Initiative Study (BIS) equation [7] for people over 70 years of age. Recently, Hans Pottel [8, 9] proposed a full age spectrum (FAS) equation that could cover all ages. Studies have shown that the FAS equation is also suitable for the Chinese general population [10, 11, 12]. Besides assessing renal function, the above-mentioned equations are also widely used to assess the risk of death, AKI, and other adverse events [13, 14, 15, 16, 17].

Xiaoyun Wu *et al*. [17] study demonstrated that the CKD-EPI equation, which was based on serum creatinine, has a better predictive power than the CG and MDRD equations on the incidence of postoperative AKI in on-pump heart surgery. However, their study did not include eGFR calculated by cystatin C. Cystatin C, a 13-kDa cysteine proteinase inhibitor protein, is freely filtered by the kidney with near-complete reabsorption and catabolism in the proximal tubule and no significant urinary excretion. Serum cystatin C is much less affected by patient characteristics such as gender, age, nutritional status, and sarcopenia than serum creatinine [18, 19, 20]. Therefore, equations developed based on cystatin may have a wide range of applications. It is therefore necessary to evaluate the prediction value of eGFR calculated by cystatin C.

With the improvement of surgical techniques, more and more patients are receiving off-pump coronary artery bypass grafting. Since the conclusions of the previous study were based on patients who underwent on-pump surgery [17, 21], it is necessary to explore the predictive power of each of the eGFR equations after off-pump surgery. The aim of our study was to compare the predictive power of preoperative eGFR calculated by each equation (including CG, MDRD, ${\mathrm{CKD-EPI}}_{\text{Creatinine}}$, ${\mathrm{CKD-EPI}}_{\text{Cystatin C}}$, ${\mathrm{FAS}}_{\text{Creatinine}}$, and ${\mathrm{FAS}}_{\text{Cystatin C}}$) for AKI after off-pump coronary artery bypass grafting.

## 2. Materials and Methods

### 2.1 Patients

**Fig. 1.**
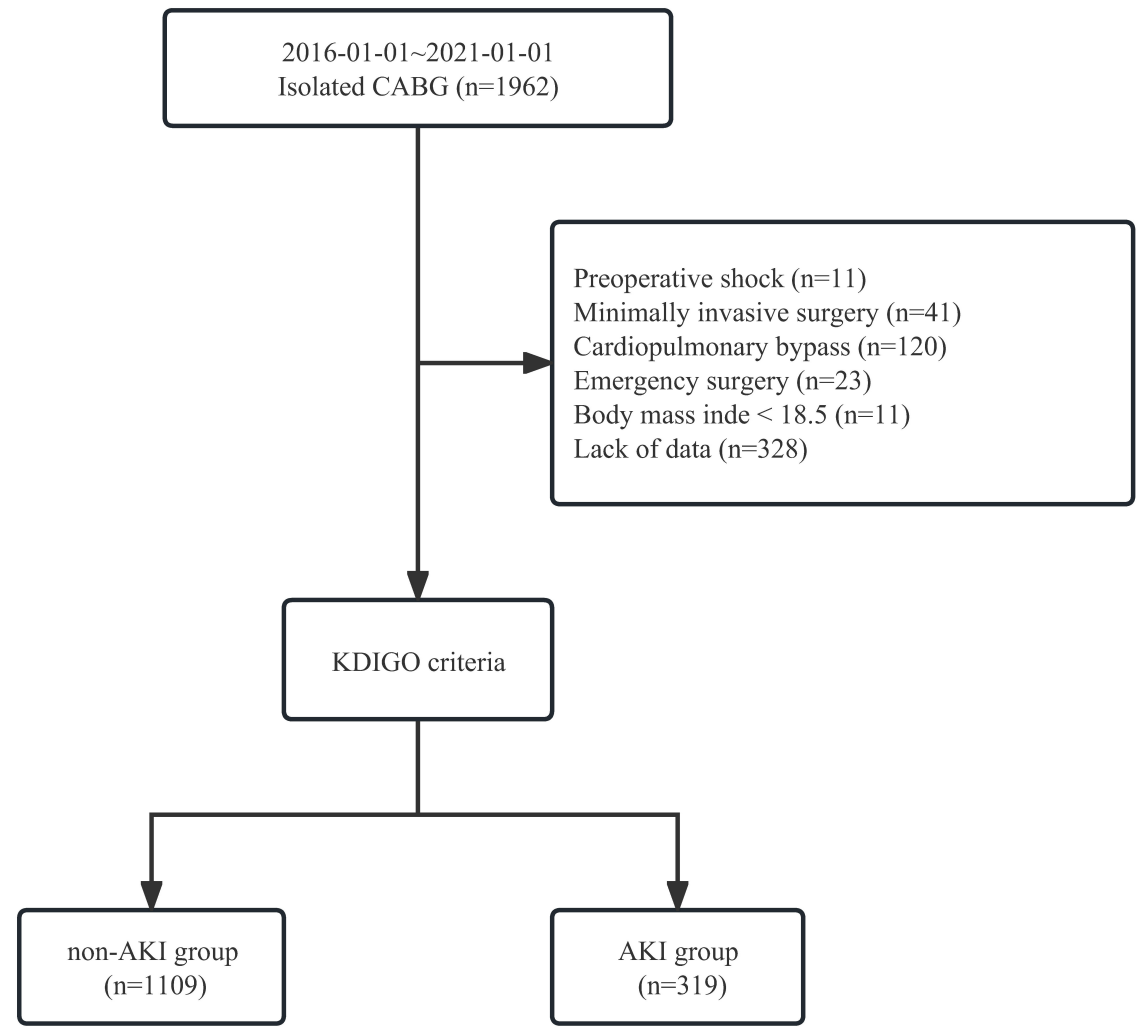
**Process of patient enrollment**. CABG, coronary artery bypass grafting; KDIGO, Kidney Disease Improving Global Outcomes; AKI, acute kidney injury.

This study was a single center, retrospective case-control study, of consecutively reviewed patients who underwent isolated coronary artery bypass grafting (CABG) at the Affiliated Hospital of Qingdao University. The study protocol was approved by the Ethics Committee of the Affiliated Hospital of Qingdao University, and informed consent was waived. The clinical data of all patients were derived from the medical scientific research data system (YIDUYUN System) of the Affiliated Hospital of Qingdao University, and the data did not contain privacy information. Laboratory samples were collected 7 days prior to surgery. Patients who met the following conditions were included: ① underwent isolated of-pump CABG in our cardiac center from January 1, 2016, to January 1, 2021; ② patients were older than 18 years. Patients with the following characteristics were excluded: ① patients with ventricular arrhythmias or cardiogenic shock before surgery (n = 11); ② underwent minimally invasive surgery (n = 41); ③ underwent cardiopulmonary bypass (n = 120); ④ emergency surgery (n = 23); ⑤ body mass index less than 18.5 (n = 11); ⑥ patients with missing data (n = 328). To avoid the effects of sarcopenia on serum creatinine levels, we excluded patients with a lower BMI. The patient enrollment process is shown in Fig. 1.

### 2.2 Definition

Six equations [3, 4, 5, 8, 9] are shown in Table 1. Postoperative AKI was diagnosed according to Kidney Disease Improving Global Outcomes (KDIGO) criteria (① urine volume <0.5 mL/kg/h for 6 h; ② an increase in serum creatinine by ≥26.5 µmol/L within 48 h; ③ an increase in serum creatinine to ≥1.5 times baseline levels, which is known or presumed to have occurred within the prior 7 days), and the occurrence of AKI within 7 days after surgery was followed. According to KDIGO’s diagnostic criteria, patients were divided into AKI group and non-AKI group. Shrunken pore syndrome (SPS) is a condition in which the eGFR based on cystatin C is significantly lower than the eGFR based on serum creatinine. According to previous studies [22, 23], the diagnostic criteria of SPS was defined when the ${\mathrm{CKD-EPI}}_{\text{Cystatin C}}$ is <70% of ${\mathrm{CKD-EPI}}_{\text{Creatinine}}$.

**Table 1.** **Details of six equations**.

Names	Levels	Equations
Cockcroft Gault		(140 – Age) × Weight / (72 × Cr) × 0.85 (if female)
MDRD		175 × Cr^ – 1.234 × Age^ – 0.179 × 0.79 (if female)
${\mathrm{CKD-EPI}}_{\text{Creatinine}}$		
	Female Cr ≤0.7	144 × (Cr/0.7)^ – 0.329 × 0.993^Age
	Female Cr >0.7	144 × (Cr/0.7)^ – 1.209 × 0.993^Age
	Male Cr ≤0.9	141 × (Cr/0.9)^ – 0.411 × 0.993^Age
	Male Cr >0.9	141 × (Cr/0.9)^ – 1.209 × 0.993^Age
${\mathrm{CKD-EPI}}_{\text{Cystatin C}}$		
	Cys ≤0.8	133 × (Cys/0.8)^ – 0.499 × 0.996^Age × 0.932 (if female)
	Cys >0.8	133 × (Cys/0.8)^ – 1.328 × 0.996^Age × 0.932 (if female)
${\mathrm{FAS}}_{\text{Creatinine}}$		
	2 ≤ Age ≤ 40 years	107.3/(Cr/Q1)
	Age >40 years	107.3/(Cr/Q1) × 0.988^(Age – 40)
${\mathrm{FAS}}_{\text{Cystatin C}}$		
	2 ≤ Age ≤ 40 years	107.3/(Cys/Q2)
	Age >40 years	107.3/(Cys/Q2) × 0.988^(Age – 40)

Note: Weight (Kg); Cr, creatinine (mg/dL), Cys, cystatin C (mg/L); Q1 = 0.70 (mg/dL) for females and Q1 = 0.90 (mg/dL) for males; Q2 = 0.82 mg/L for ages <70 years and Q2 = 0.95 mg/L for older ages. MDRD, modification of diet in renal disease; CKD-EPI, chronic kidney disease-epidemiology; FAS, full age spectrum.

### 2.3 Procedures and Postoperative Management

Oxygen inhalation and breathing training were given to all patients after admission. Anti-platelet therapy, myocardial nutrition (such as CoenzyMe Q10, Trimetazidine and Potassium Magnesium Aspartate Tablets), and other treatments were given according to the patient’s condition. All patients underwent a sternotomy, and received heparin (1 mg/kg). During surgery, the left anterior descending artery was grafted first, followed by the diagonal artery or obtuse marginal artery, and finally the posterior descending artery and the right posterior lateral artery.

### 2.4 Statistical Analysis

All continuous variables were described by the mean ± standard deviation, categorical variables are expressed in absolute numbers (percentages). Differences in basic information and biochemical testing between the AKI group and non-AKI group were compared using the student’s *t*-test or chi-square test, respectively. A structured adjustment scheme was used to control for confounders: Model 1 unadjusted. Model 2 adjusted for age and sex. Model 3 adjusted for age, sex, heart failure, hypertension, chronic kidney disease, uric acid, ejection fraction, shrunken pore syndrome, and diuretics therapy before surgery. A receiver operating characteristic (ROC) curve was used to evaluate the ability of each eGFRs to correctly distinguish AKI. The diagnostic accuracy in predicting AKI was examined through the area under the receiver operating characteristic curve (AUC) with a 95% confidence interval. Differences between the AUC of evaluated variables were performed with a non-parametric approach (DeLong test). In addition, we performed further exploratory stratified analyses for sex and age. Finally, we examined the nonlinear correlation between eGFRs and odd ratios of AKI with restricted cubic splines. The calibration curve was used to compare the predicted probability of AKI by eGFRs with the observed probability of AKI. *p* < 0.05 was considered statistically significant. SPSS 26.0 (IBM Corp., Armonk, NY, USA), GraphPad Prism 9.0 (GraphPad Software, Inc., San Diego, CA, USA), and R-project 4.1.2 (The R Foundation for Statistical Computing, Vienna, Austria) were used for the analyses.

## 3. Results

### 3.1 General Data Analysis

In the total cohort of 1428 patients, the mean age was 63.7 years, and 74.4% were male. The prevalence of hypertension, diabetes and shrunken pore syndrome was 72.8%, 39.5% and 13.3%, respectively. In this cohort, 25.5% (319/1428) of patients developed postoperative AKI. The mean eGFR for the entire study are CG (84.0 ± 22.7 mL/min/1.73 $m^{2}$), MDRD (95.5 ± 26.1 mL/min/1.73 $m^{2}$), ${\mathrm{CKD-EPI}}_{\text{crea}}$ (84.9 ± 18.7 mL/min/1.73 $m^{2}$), ${\mathrm{CKD-EPI}}_{\text{cys}}$ (80.0 ± 21.7 mL/min/1.73 $m^{2}$), ${\mathrm{FAS}}_{\text{crea}}$ (82.9 ± 23.7 mL/min/1.73 $m^{2}$), and ${\mathrm{FAS}}_{\text{cys}}$ (72.6 ± 18.4 mL/min/1.73 $m^{2}$). Perioperative patient characteristics are shown in Table 2.

**Table 2.** **Baseline Characteristics of the Patients**.

Variables	Overall	Non-AKI	AKI	*p* value
	n = 1428	n = 1109	n = 319	
Age (year, mean ± SD)	63.7 (8.3)	63.0 (8.2)	66.2 (7.9)	<0.001
Sex (male, %)	1063 (74.4)	828 (74.7)	235 (73.7)	0.775
High (cm, mean ± SD)	167.0 (7.5)	167.1 (7.3)	166.4 (7.8)	0.119
Weight (Kg, mean ± SD)	71.8 (11.1)	71.9 (11.2)	71.2 (10.5)	0.291
Body mass index (${\mathrm{kg/m}}^{2}$, mean ± SD)	25.7 (3.1)	25.7 (3.2)	25.7 (3.1)	0.949
Smoking (%)	631 (44.2)	489 (44.1)	142 (44.5)	0.945
Acute coronary syndrome (%)	221 (15.5)	172 (15.5)	49 (15.4)	1
Heart failure (%)	57 (4.0)	34 (3.1)	23 (7.2)	0.002
Coronary stent (%)	182 (12.7)	141 (12.7)	41 (12.9)	1
Hypertensions (%)	1040 (72.8)	787 (71.0)	253 (79.3)	0.004
Diabetes mellitus (%)	564 (39.5)	431 (38.9)	133 (41.7)	0.398
Stroke (%)	222 (15.5)	166 (15.0)	56 (17.6)	0.256
Peripheral vascular disease (%)	42 (2.9)	32 (2.9)	10 (3.1)	0.851
Chronic obstructive pulmonary disease (%)	28 (2.0)	21 (1.9)	7 (2.2)	0.818
Chronic kidney disease (%)	13 (0.9)	7 (0.6)	6 (1.9)	0.049
Shrunken pore syndrome (%)	190 (13.3)	113 (10.2)	77 (24.1)	<0.001
Creatinine (mg/L, mean ± SD)	79.9 (36.1)	75.4 (18.8)	95.6 (65.5)	<0.001
Cystatin C (mg/L, mean ± SD)	1.0 (0.4)	0.9 (0.2)	1.3 (0.6)	<0.001
Triglyceride (mmol/L, mean ± SD)	1.7 (1.3)	1.7 (1.2)	1.8 (1.6)	0.266
Cholesterol (mmol/L, mean ± SD)	4.2 (1.3)	4.3 (1.3)	4.1 (1.4)	0.016
High-density lipoproteins (mmol/L, mean ± SD)	1.1 (0.3)	1.2 (0.3)	1.1 (0.3)	0.014
Low-density lipoproteins (mmol/L, mean ± SD)	2.5 (0.9)	2.6 (1.0)	2.5 (0.9)	0.32
Lipoprotein a (mg/L, median IQR)	208.4 [110.0, 406.1]	208.2 [110.0, 402.0]	211.0 [110.7, 427.0]	0.849
Apolipoprotein AI (g/L, mean ± SD)	1.2 (0.2)	1.2 (0.2)	1.2 (0.2)	0.094
Apolipoprotein B (g/L, mean ± SD)	0.9 (0.3)	0.9 (0.3)	0.8 (0.3)	0.226
Fasting blood glucose (mmol/L, mean ± SD)	6.3 (2.5)	6.2 (2.4)	6.5 (2.7)	0.144
Uric acid (µmol/L, mean ± SD)	335.8 (92.8)	330.8 (87.5)	353.4 (107.5)	<0.001
Hemoglobin (g/L, mean ± SD)	131.7 (17.4)	132.5 (17.0)	129.0 (18.3)	0.002
Platelet (${\mathrm{10}}^{9}$/L, mean ± SD)	219.2 (63.0)	218.8 (63.3)	220.5 (61.9)	0.667
High-sensitivity troponin I (ng/mL, median IQR)	0.014 (0.008, 0.029)	0.014 (0.008, 0.027)	0.017 (0.010, 0.041)	<0.001
Ejection Fraction (%, mean ± SD)	57.5 (7.1)	57.9 (6.9)	56.2 (7.5)	<0.001
Angiotensin converting enzyme inhibitor (%)	271 (19.0)	203 (18.3)	68 (21.3)	0.259
Angiotensin receptor blocker (%)	494 (34.6)	378 (34.1)	116 (36.4)	0.492
Beta receptor blocker (%)	1044 (73.1)	805 (72.6)	239 (74.9)	0.449
Calcium channel blocker (%)	521 (36.5)	389 (35.1)	132 (41.4)	0.046
Statin (%)	1134 (79.4)	893 (80.5)	241 (75.5)	0.063
Digoxin (%)	65 (4.6)	47 (4.2)	18 (5.6)	0.288
Diuretics (%)	525 (36.8)	368 (33.2)	157 (49.2)	<0.001
Surgical time (min, mean ± SD)	261.5 (78.4)	261.3 (79.9)	262.2 (73.1)	0.86
Left internal thoracic artery (%)	1290 (90.3)	1004 (90.5)	286 (89.7)	0.719
Incomplete revascularization (%)	34 (2.4)	25 (2.3)	9 (2.8)	0.535
eGFR (mL/min/1.73 $m^{2}$, mean ± SD)				
Cockcroft Gault	84.0 (22.7)	86.4 (21.3)	75.5 (25.6)	<0.001
MDRD	95.5 (26.1)	98.1 (24.0)	86.3 (30.7)	<0.001
${\mathrm{CKD-EPI}}_{\text{crea}}$	84.9 (18.7)	88.7 (15.9)	71.8 (21.7)	<0.001
${\mathrm{CKD-EPI}}_{\text{cys}}$	80.0 (21.7)	84.4 (19.4)	64.8 (22.3)	<0.001
${\mathrm{FAS}}_{\text{crea}}$	82.9 (23.7)	85.7 (22.8)	72.9 (24.3)	<0.001
${\mathrm{FAS}}_{\text{cys}}$	72.6 (18.4)	76.2 (17.1)	60.2 (17.3)	<0.001

AKI, acute kidney injury; SD, standard deviation; eGFR, estimated glomerular filtration rate; MDRD, modification of diet in renal disease; CKD-EPI, chronic kidney disease-epidemiology; FAS, full age spectrum; IQR, inter-quartile range.

### 3.2 Differences between AKI and Non-AKI Groups

**Fig. 2.**
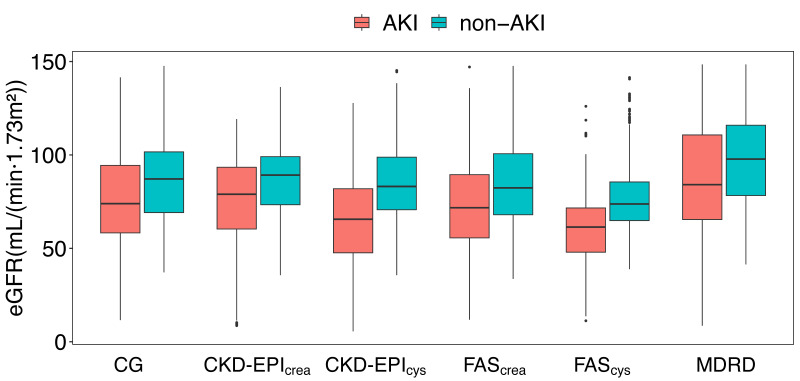
**The distribution of eGFR levels in AKI and non-AKI groups**. AKI, acute kidney injury; eGFR, estimated glomerular filtration rate; MDRD, modification of diet in renal disease; CKD-EPI, chronic kidney disease-epidemiology; FAS, full age spectrum; CG, Cockcroft Gault.

Patients with AKI were more likely to be older, and had an increased incidence of heart failure, hypertension, and chronic kidney disease. Patients in the AKI group were more likely to have received diuretics, to have a lower left ventricular ejection fraction and eGFR. The AKI group had a higher prevalence of SPS than the non-AKI group. The distribution of eGFR levels in AKI and non-AKI groups are shown in Fig. 2. These values were calculated by six equations.

### 3.3 Multivariate Logistic Regression Analysis

**Fig. 3.**
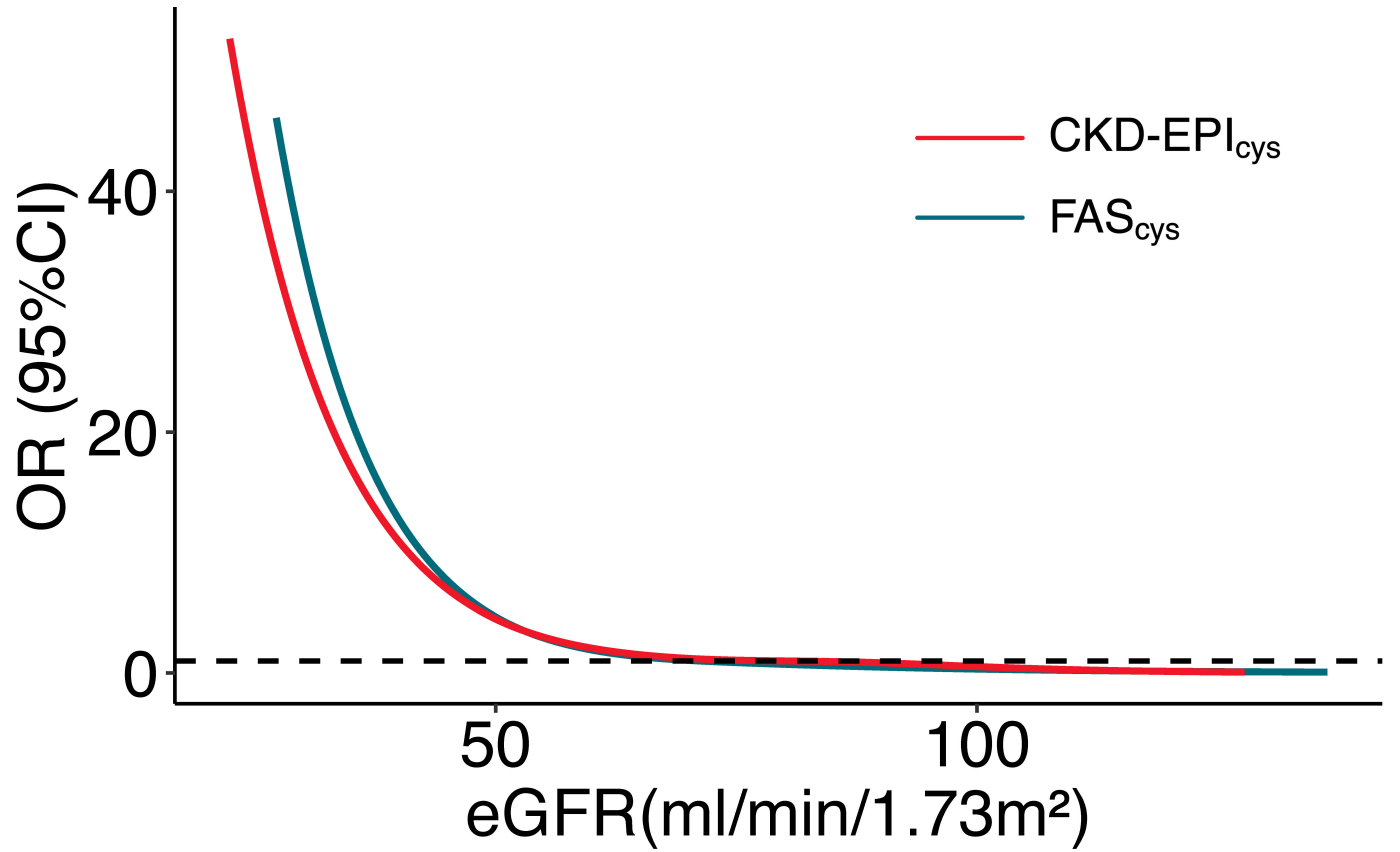
**The correlation between OR and eGFR levels**. CKD-EPI, chronic kidney disease-epidemiology; FAS, full age spectrum; OR, odds ratio; eGFR, estimated glomerular filtration rate; CI, confidence interval.

The odds ratios (ORs) and 95% confidence interval (CI) of each eGFRs are presented in Table 3. The results of Univariate logistic regression analyses indicated that all eGFR equations were significantly correlated with postoperative AKI (all *p* < 0.001). Model 1 was unadjusted; Model 2 was adjusted for age and sex; Model 3 was adjusted for age, sex, heart failure, hypertension, chronic kidney disease, uric acid, ejection fraction, shrunken pore syndrome, and diuretics therapy before surgery. After adjusted confounders, all equations had an effective power to predict postoperative AKI. Among them, ${\mathrm{CKD-EPI}}_{\text{cys}}$ and ${\mathrm{FAS}}_{\text{cys}}$ equations have the smallest OR values. We also examined the nonlinear correlation between eGFRs calculated by ${\mathrm{FAS}}_{\text{cys}}$ and ${\mathrm{CKD-EPI}}_{\text{cys}}$ and odd ratio of AKI with RCS. The risk of AKI dramatically increased as eGFRs decreased. The correlation between eGFRs of ${\mathrm{CKD-EPI}}_{\text{cys}}$ and ${\mathrm{FAS}}_{\text{cys}}$ equations and odd ratio of AKI is shown in Fig. 3.

**Table 3.** **Logistic regression analysis in three models**.

Equations	Model 1		Model 2		Model 3	
	OR (95% CI)	*p* value	OR (95% CI)	*p* value	OR (95% CI)	*p* value
Cockcroft Gault	0.979 (0.973–0.984)	<0.001	0.982 (0.976–0.988)	<0.001	0.983 (0.977–0.99)	<0.001
MDRD	0.982 (0.977–0.987)	<0.001	0.983 (0.978–0.988)	<0.001	0.983 (0.978–0.989)	<0.001
${\mathrm{CKD-EPI}}_{\text{crea}}$	0.969 (0.962–0.975)	<0.001	0.971 (0.964–0.978)	<0.001	0.97 (0.962–0.978)	<0.001
${\mathrm{CKD-EPI}}_{\text{cys}}$	0.953 (0.947–0.96)	<0.001	0.955 (0.948–0.962)	<0.001	0.955 (0.946–0.963)	<0.001
${\mathrm{FAS}}_{\text{crea}}$	0.975 (0.969–0.981)	<0.001	0.978 (0.972–0.984)	<0.001	0.978 (0.971–0.985)	<0.001
${\mathrm{FAS}}_{\text{cys}}$	0.94 (0.931–0.949)	<0.001	0.94 (0.931–0.95)	<0.001	0.941 (0.93–0.953)	<0.001

MDRD, modification of diet in renal disease; CKD-EPI, chronic kidney disease-epidemiology; FAS, full age spectrum; OR, odds ratio; CI, confidence interval.

### 3.4 ROC Analysis

**Fig. 4.**
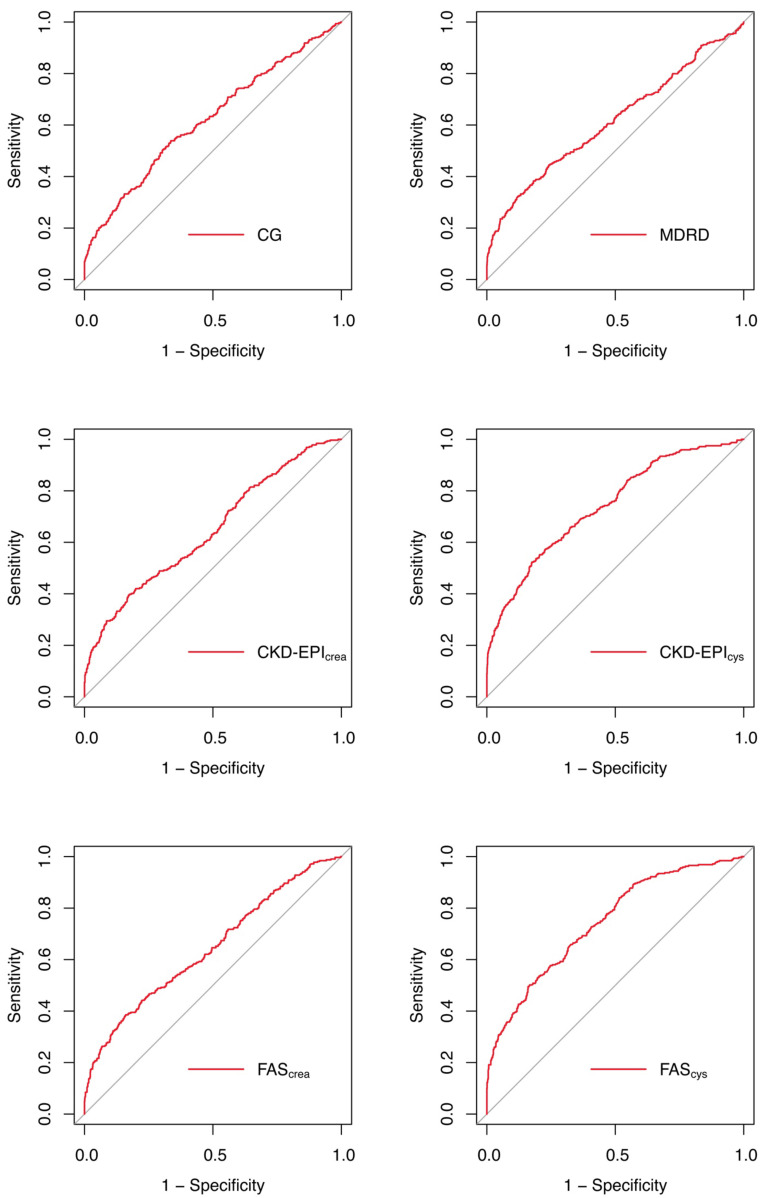
**ROC curves of each equations in the total cohort**. ROC, receiver operating characteristic; CG, Cockcroft Gault; MDRD, modification of diet in renal disease; CKD-EPI, chronic kidney disease-epidemiology; FAS, full age spectrum.

ROC analysis and subgroup analysis of six eGFR equations are demonstrated in Table 4. The ROC curve of the total cohort is shown in Fig. 4. In the ability to predict postoperative AKI, ${\mathrm{FAS}}_{\text{cys}}$ and ${\mathrm{CKD-EPI}}_{\text{cys}}$ equations had the higher AUC (0.739, 95% CI 0.708–0.771 and 0.744, 95% CI 0.713–0.774), followed by, ${\mathrm{CKD-EPI}}_{\text{crea}}$, ${\mathrm{FAS}}_{\text{crea}}$, CG, and MDRD equations, with the AUCs ranging from 0.614 to 0.643 in the total study population.

**Table 4.** **ROC analysis and ROC analysis in the subgroups**.

Equations		AUC	95% CI	*p* value
Total cohort	n = 1428			
	Cockcroft Gault	0.621	0.584–0.657	<0.001
	MDRD	0.614	0.576–0.652	<0.001
	${\mathrm{CKD-EPI}}_{\text{crea}}$	0.643	0.608–0.678	<0.001
	${\mathrm{CKD-EPI}}_{\text{cys}}$	0.739	0.708–0.771	<0.001
	${\mathrm{FAS}}_{\text{crea}}$	0.643	0.607–0.678	<0.001
	${\mathrm{FAS}}_{\text{cys}}$	0.744	0.713–0.774	<0.001
Male	n = 1063			
	Cockcroft Gault	0.638	0.596–0.68	<0.001
	MDRD	0.629	0.585–0.673	<0.001
	${\mathrm{CKD-EPI}}_{\text{crea}}$	0.661	0.62–0.701	<0.001
	${\mathrm{CKD-EPI}}_{\text{cys}}$	0.742	0.705–0.779	<0.001
	${\mathrm{FAS}}_{\text{crea}}$	0.66	0.619–0.701	<0.001
	${\mathrm{FAS}}_{\text{cys}}$	0.752	0.716–0.788	<0.001
Female	n = 365			
	Cockcroft Gault	0.575	0.501–0.649	0.037
	MDRD	0.569	0.495–0.642	0.057
	${\mathrm{CKD-EPI}}_{\text{crea}}$	0.588	0.517–0.659	0.014
	${\mathrm{CKD-EPI}}_{\text{cys}}$	0.728	0.668–0.787	<0.001
	${\mathrm{FAS}}_{\text{crea}}$	0.591	0.518–0.663	0.012
	${\mathrm{FAS}}_{\text{cys}}$	0.72	0.661–0.78	<0.001
Age ≥65	n = 698			
	Cockcroft Gault	0.565	0.517–0.613	0.006
	MDRD	0.568	0.519–0.618	0.004
	${\mathrm{CKD-EPI}}_{\text{crea}}$	0.577	0.528–0.625	0.001
	${\mathrm{CKD-EPI}}_{\text{cys}}$	0.674	0.628–0.72	<0.001
	${\mathrm{FAS}}_{\text{crea}}$	0.578	0.53–0.627	0.001
	${\mathrm{FAS}}_{\text{cys}}$	0.675	0.629–0.72	<0.001
Age <65	n = 730			
	Cockcroft Gault	0.627	0.565–0.69	<0.001
	MDRD	0.675	0.616–0.734	<0.001
	${\mathrm{CKD-EPI}}_{\text{crea}}$	0.683	0.627–0.74	<0.001
	${\mathrm{CKD-EPI}}_{\text{cys}}$	0.784	0.738–0.83	<0.001
	${\mathrm{FAS}}_{\text{crea}}$	0.68	0.623–0.737	<0.001
	${\mathrm{FAS}}_{\text{cys}}$	0.783	0.737–0.83	<0.001

ROC, receiver operating characteristic; AUC, area under the receiver operating characteristic curve; CI, confidence interval; MDRD, modification of diet in renal disease; CKD-EPI, chronic kidney disease-epidemiology; FAS, full age spectrum.

In the age and sex subgroups, we found that ${\mathrm{FAS}}_{\text{cys}}$ and ${\mathrm{CKD-EPI}}_{\text{cys}}$equations still showed higher diagnostic values than the rest of the equations. After DeLong’s test, ${\mathrm{FAS}}_{\text{cys}}$ equation showed no difference compared with ${\mathrm{CKD-EPI}}_{\text{cys}}$ in any subgroup (all *p* > 0.05). The best diagnostic performance of ${\mathrm{FAS}}_{\text{cys}}$ (0.783, 95% CI 0.737–0.83) and ${\mathrm{CKD-EPI}}_{\text{cys}}$ (0.784, 95% CI 0.738–0.83) were found in patients younger than 65 years, when compared with ${\mathrm{CKD-EPI}}_{\text{crea}}$ (0.683, 95% CI 0.627–0.74), ${\mathrm{FAS}}_{\text{crea}}$ (0.68, 95% CI 0.623–0.737), CG (0.627, 95% CI 0.565–0.69) and MDRD (0.675, 95% CI 0.616–0.734). In older patients, AUCs of the equations (including ${\mathrm{CKD-EPI}}_{\text{crea}}$, ${\mathrm{FAS}}_{\text{crea}}$, CG, and MDRD) were less than 0.6, hence these equations could not be used alone to predict postoperative AKI. In older patients, the predictive power of ${\mathrm{FAS}}_{\text{cys}}$ and ${\mathrm{CKD-EPI}}_{\text{cys}}$ equations were significantly attenuated, and AUCs of two equations were less than 0.7.

### 3.5 Calibration Curves

**Fig. 5.**
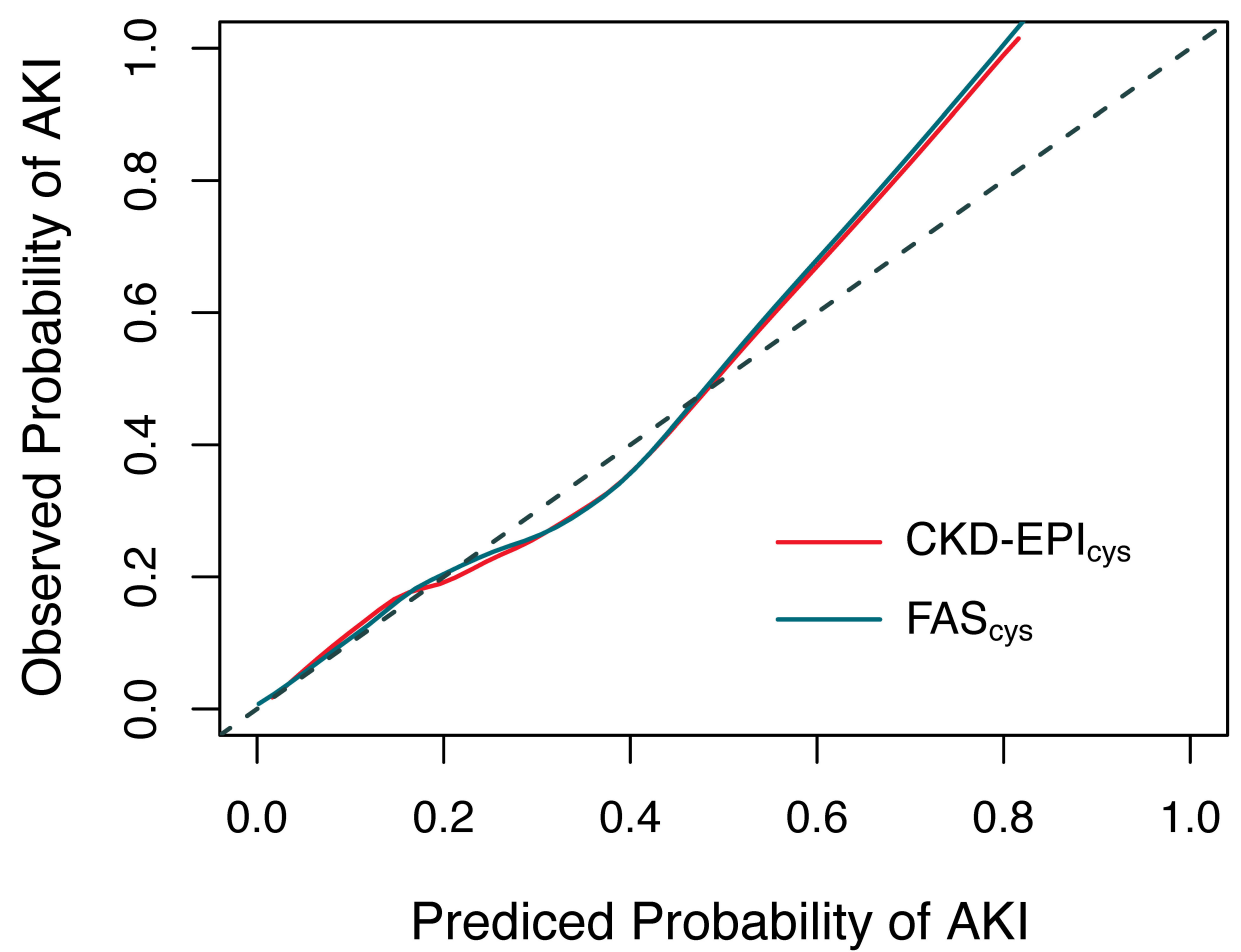
**The calibration curves of ${\mathrm{FAS}}_{\text{Cystatin C}}$ and ${\mathrm{CKD-EPI}}_{\text{Cystatin C}}$**. AKI, acute kidney injury; CKD-EPI, chronic kidney disease-epidemiology; FAS, full age spectrum.

The calibration curves showed that there is a good agreement between the predicted probability by two equations (${\mathrm{FAS}}_{\text{cys}}$ and ${\mathrm{CKD-EPI}}_{\text{cys}}$) and the observed probability of AKI. Therefore, using the two equations to predict AKI would not overestimate or underestimate the risk of postoperative AKI for patients undergoing off-pump coronary surgery. The calibration curves are shown in Fig. 5.

## 4. Discussion

This study found that preoperative eGFRs calculated based on cystatin C have more accurate predictive power for AKI after off-pump CABG. Moreover, in patients younger than 65 years, all equations had better performance than patients over 65 years. Compared with each eGFR, we also found that the average value of eGFR calculated by the FAS equation based on cystatin C was the lowest, while the average value of the MDRD equation was the highest. The predictive power of preoperative eGFR based on cystatin C was better than creatinine. This phenomenon may be related to the following two reasons: ① cystatin C is better than creatinine in evaluating renal function. It was much less affected by patient characteristics, such as gender, age, body size and composition, and nutritional status [18, 19, 20]. ② Due to SPS, eGFRs calculated by the creatinine could be overestimated. In this study, the incidence of SPS was significantly higher in the AKI group than in the non-AKI group. Therefore, we suggest using equations based on cystatin C to calculate eGFR [22, 23].

Xiaoyun Wu *et al*. [17] compared the predictive value of eGFRs after cardiac surgery. The main conclusion of their paper is that the CKD-EPI equation has a better predictive effect than the CG and MDRD equations. Additionally, the body surface area adjusted CG equation performed better in their cohort. In the comparison of the predictive power of CKD-EPI, CG, and MDRD equations, the results of our study are consistent with their conclusions. It is important to note that the decrease in perfusion pressure during on-pump surgery can alter renal function. Hence, the occurrence of postoperative AKI, in their study, is more than 2-fold of our cohort. Differences were also seen in sample size, average age, and the incidence of males/females. Wu’s study also explored the predictive effect of eGFR within 2 days after surgery. Patients were being treated in the cardiac intensive care unit at this time. Hence, AKI can be directly and conveniently diagnosed based on urine volume or creatinine levels. Therefore, it is not necessary to calculate eGFR to predict AKI alone. In summary, Wu’s conclusion cannot be directly applied to all cardiac surgery patients.

In the subgroup analysis, we also found that the predictive effect of the eGFR equations was enhanced in patients less than 65 years. This phenomenon may be related to the patient’s condition when the equation was established. Younger patients have a lower incidence of heart failure, atrial fibrillation, and other co-morbidities. Therefore, renal function may have a greater impact on postoperative AKI in younger patients. In other studies, the equations were more accuracy for the diagnosis and stratification in patients with advanced kidney disease or older age [24, 25]. But none of the patients in these studies underwent cardiac surgery.

Notably, in our study, eGFR based cystatin C had better predictive power in the diabetic population. Studies have also demonstrated that serum cystatin C has better predictive power for adverse outcomes in diabetic patients. Kati Jarvela *et al*.’s [26] study prospectively enrolled 200 patients who underwent elective CABG, and measured their serum creatinine and cystatin C levels. Study point out that cystatin C and cystatin C-based estimation of GFR may be useful and more sensitive than creatinine in detecting mild acute renal insufficiency in diabetic patients. In addition, Caroline Pereira Domingueti and colleagues [27] also demonstrated that cystatin C-based equations present the best accuracy to detect macroalbuminuria in Cystatin type 1 diabetes mellitus patients. This phenomenon may be related to the fact that diabetes not only increases serum cystatin levels, but also significantly increases the risk of postoperative AKI [26, 28].

By comparing each equations, we identified two equations that were suitable for preoperative assessment of renal function. Accurate assessment of preoperative renal function not only could predict the risk of postoperative AKI, but also be used to guide preoperative clinical strategy to avoid AKI. In addition, AKI is influenced by multiple perioperative risk factors, which may explain the limited predictive power of eGFR. Despite improvement in bias compared with equations based on creatinine, some studies suggest that a few biases remain in equations based on cystatin C, especially in patients who are chronically ill. Among them, measured GFR may be necessary for the accurate assessment of GFR in these populations [20, 29]. On the other hand, some studies found that further modification and the addition of the Chinese racial factor could improve the predictive and stratification ability of preoperative eGFR [12, 30]. Hence, further research is needed.

### Limitation

This study was a single-center, retrospective, cohort study. Therefore, various biases in retrospective studies also exist in this study. Due to the limitations of the study design, measurement of GFR, a gold standard, could not be performed, so the results need to be further verified in large-scale, multi-center prospective studies.

## 5. Conclusions

Preoperative eGFR calculated by ${\mathrm{FAS}}_{\text{cys}}$ and ${\mathrm{CKD-EPI}}_{\text{cys}}$ equations have better performance in predicting AKI after off-pump CABG than other equations, especially in diabetics.

## Data Availability

The data analyzed in this study are available from the corresponding author or first author upon reasonable request.
